# Exercise Addiction During the COVID-19 Pandemic: an International Study Confirming the Need for Considering Passion and Perfectionism

**DOI:** 10.1007/s11469-020-00433-7

**Published:** 2020-12-03

**Authors:** Ricardo de la Vega, Lucia Jiménez Almendros, Roberto Ruíz Barquín, Szilvia Boros, Zsolt Demetrovics, Attila Szabo

**Affiliations:** 1 Department of Physical Education, Sport and Human Movement, Autonomous University of Madrid, Madrid, Spain; 2 Department of Developmental and Educational Psychology, Autonomous University of Madrid, Madrid, Spain; 3 Institute of Health Promotion and Sport Sciences, ELTE Eötvös Loránd University, Bogdánfy u. 10/B, Budapest, 1117 Hungary; 4 Institute of Psychology, ELTE Eötvös Loránd University, Budapest, Hungary

**Keywords:** Exercise dependence, Individual sport, Reason for exercise, Team sport, Training

## Abstract

Various levels of lockdown due to COVID-19 limit people’s habitual physical activity. Individuals addicted to exercise, health-oriented, and team-exercisers could be the most affected. We examined the COVID-19-related changes in exercise volume in 1079 exercisers from eight Spanish-speaking nations based on exercise addiction categories, primary reasons for exercise, and forms of exercise. The COVID-19-related decrease in exercise volume was 49.24% in the sample. The proportion of the risk of exercise addiction was 15.2%. Most (81.7%) of the participants exercised for a health-related reason. These exercisers reported lesser decrease in their exercise volume than those exercising for social reasons. The risk of exercise addiction was inversely related to changes in exercise volume, but after controlling for passion and perfectionism the relationship vanished. The reported effect of COVID-19 on training did not differ between the exercise addiction groups. The findings also confirm that exercise addiction research should control for passion and perfectionism.

A physically active lifestyle has physical (Lee et al. 2011) and mental health benefits (Clow and Edmunds 2013). Most often people engage in physical activity for a health reason, such as coping with stress (Berczik et al. 2012; Szabo et al. 2019). In certain cases, the habitual physical activity could become compulsive (Stevens et al. 2013), which stems from tolerance (a natural training effect) and urges the person to progressively increase her/his exercise to achieve the same benefits as before. The craving for more and more exercise may result in loss of control, and from that point on the behavior is no longer “healthy” (Szabo 2010). When a person loses control over her/his physical activity a dysfunctional behavior, known as “exercise addiction,” may be observed (Szabo 2010; Szabo et al. 2015). While this morbidity is relatively rare (i.e., ≈ 3.7%) as based on a recent meta-analysis (Trott et al. 2020), there are about 1000 published scholastic articles in the field (Szabo and Kovacsik 2019). The keen interest in the topic was only recently justified by Juwono and Szabo (2020) by revealing that there might be significantly more than the currently estimated cases of exercise addiction.

One overt sign of exercise addiction is the very high or exaggerated amount of training. However, MacLaren and Best (2007) noted that while exercise addiction is connected to high exercise volume, the latter is not evidence for addiction. Indeed, passion for exercise characterized by the love for the physical activity that one finds appealing, salient, and keenly invests time and energy into it (Vallerand et al. 2006) may be reflected in high volumes of exercise. Vallerand et al. (2003) have suggested that passion can be harmonious (HP) and obsessive (OP). The former is manifested when the beloved activity is autonomously internalized and the individual engages in it with flexibility (Vallerand et al. 2003; Vallerand et al. 2006). On the contrary, OP is manifested when the person internalizes the adopted activity in a controlled way and the activity is rigidly controlled (Vallerand et al. 2003). Further, the obsessively passionate person attaches importance to the activity’s corollaries, such as self-esteem or escape from stress, which renders it difficult to control or to terminate the passionate activity (Vallerand 2010). There is commonality between exercise addiction and passion. Indeed, OP explains a large amount of variance in exercise addiction (Kovacsik et al. 2019, 2020). The shared variance is more than twice as large in individual than in team exercisers (Kovacsik et al. 2020). Passion mediates the association between exercise addiction and exercise volume. In fact, it may wipe out the connection between the two (Szabo and Kovacsik 2019). Therefore, it was suggested that in studying exercise addiction, researchers must control for the effects of passion (Szabo and Kovacsik 2019). Indeed, these authors questioned the validity of the results of all earlier studies on exercise addiction that did not control for passion.

Apart from the strong link between exercise addiction and passion, research shows that perfectionism is associated with both (Bircher et al. 2017; Curran et al. 2014). Schipfer and Stoll (2015) in their Exercise-Addiction/Exercise-Commitment-Model (EACOM) suggested that passion and perfectionism might moderate exercise addiction. They produced preliminary evidence showing that certain aspects of both passion and perfectionism do indeed moderate exercise addiction. Thus, research on exercise addiction should not only control for passion but also for perfectionism.

The COVID-19 affects the whole world. Those who exercise in large volumes, including those at risk of exercise addiction and group/team exercisers, are forced to cut down on their exercise. However, individuals addicted to exercise will presumably find alternative means of training, including home or individual outdoor exercises that are permitted during the lockdown. Similarly, individuals who exercise for a health, or a “therapeutic” reason, may exert extra effort in trying not to cut down their volume of exercise, or otherwise they may feel loss of control over the health ailment for which they exercise (Szabo et al. 2019). Considering the interactional model for exercise addiction (Egorov and Szabo 2013), individuals who get addicted to exercise have a therapeutic (health) primary motive related to coping with life difficulties. Therefore, people at high risk of exercise addiction, exercising for a health reason, may not be able to cut down their exercise volume. Further, individuals addicted to exercise in team or a group exercises might be forced to decrease their exercise volume during the COVID-19 pandemic, because the unavailability of open facilities and absence of other group members. Consequently, it is of interest to investigate how those at risk of exercise addiction cope with the COVID-19 situation in terms of exercise volume and perceived effect of the COVID-19 pandemic on their exercise training regimen. It is also of interest to further investigate this question in individuals at risk of exercise addiction who exercise admittedly for therapeutic reason(s) and those who train in group or team settings.

The purpose of the study was to investigate changes in exercise patterns in the context of the risk of exercise addiction during early stages of the COVID-19 pandemic in eight Spanish-speaking nations, while also considering the roles of passion and perfectionism. Specifically, we were interested in examining the following: (1) the prevalence of the risk of exercise addiction in the sample; (2) the distribution of the primary exercise motive (i.e., (a)) health-, (b) skill-, and (c) social) in three exercise addiction risk groups (i.e., (a)) those at risk of exercise addiction [strong symptoms], (b) symptomatic [mild symptoms], and (c) asymptomatic [no symptoms]); (3) the change in exercise volume, in response to the COVID-19 pandemic in the three exercise addiction risk groups by considering their primary reason for exercise; (4) the relationship between the change in exercise volume during the COVID-19 pandemic and exercise addiction risk scores; (5) the change in exercise volume in response to the COVID-19 pandemic in three exercise addiction groups in the context of team or individual exercises; and (6) the perceived effect of the COVID-19 situation on the habitual exercise training regimen in the three exercise addiction risk groups.

Apart from the first question that was exploratory, we posed five hypotheses: (1) the primary motives for exercise will differ between the three exercise addiction risk groups; (2) those at high risk of exercise addiction having a primary therapeutic motive for exercise will exhibit less change in exercise volume due to the COVID-19 situation than those who are at lower risk and exercise for other than health reasons; (3) a negative relationship will emerge between the exercise addiction risk scores and the change in exercise volume during the COVID-19 pandemic; (4) team exercisers in three exercise addiction risk categories will demonstrate greater change (decrease) in exercise volume than individual exercisers during the COVID-19 pandemic (due to lack of teammates and closed facilities); and (5) those at risk of exercise addiction will report greater negative impact of the COVID-19 pandemic in their training schedules than symptomatic or asymptomatic exercisers. Due to the very novelty of the COVID-19 literature, little or no background literature is available on the connection between the pandemic and the dependent measures. Therefore, the current hypotheses were generated primarily by relying on “common sense” or rational expectations. However, to investigate the claim of Szabo and Kovacsik et al. (2019) that the nearly 1000 published papers in the field of exercise addiction, which did not control for passion, may have reported unreliable results, we double tested our hypotheses, once without including passion in the model and one more time by controlling for passion. Expanding further the work of Szabo and Kovacsik et al. (2019) and accounting for EACOM (Schipfer and Stoll 2015), we also added perfectionism to the second model (conditionally, based upon a significant relationship between exercise addiction risk scores and perfectionism).

## Materials and Methods

### Participants

Participants were recruited via social media from eight Spanish speaking countries: Argentina (*n* = 102), Chile (*n* = 95), Costa Rica (*n* = 93), Ecuador (*n* = 99), Honduras (*n* = 77), Mexico (*n* = 96), Spain (*n* = 422), and Uruguay (*n* = 95). The total number of eligible participants was 1079 (52% men). Their mean age was 32.88 (± SD = 11.73) years, ranging from 18 to 75 years. More than half (53.1%) of the sample was involved in team/group exercises while the rest exercised on an individual basis. The majority (81.7%) exercised primarily for a health-related reason, 11.7% for a skill-related reason, and 6.6% for a social reason. While most participants were involved in one form of exercise, 24% of them reported practicing more than one exercise.

### Materials

A battery of questionnaires, comprised of demographic and exercise habits questions as well as three psychometric instruments, was used in the current study. The demographic questions only asked for age and gender. Questions about exercise habits gauged the main reason for exercise behavior (i.e., health, skill, or social relation related), the weekly amount of exercise (hours per week) before and during the COVID-19 pandemic, and form of exercise (individual or team). A single item 11-point Likert scale was used for assessing the perceived impact of COVID-19 on respondents’ training regimen. Additionally, three psychometric instruments were employed for measuring the risk of exercise addiction, passion, and perfectionism.

The Exercise Addiction Inventory (EAI; Terry et al. 2004) was used to assess the risk of exercise addiction (EA). An example statement is “Exercise is the most important thing in my life.” The EAI has six items which are rated on a 5-point Likert scale, ranging from “strongly disagree” to “strongly agree.” Higher scores indicate higher risk of EA. The EAI was presented with good psychometric properties (Griffiths et al. 2015; Terry et al. 2004). In the current research, we used the Spanish version of the scale (Sicilia et al. 2013). The internal reliability of the Spanish EAI is (Cronbach’s *α*) 0.70 (Griffiths et al. 2015; Sicilia et al. 2013). In the current inquiry, the internal reliability of the EAI was 0.65, which fits the lower level of the *α* range (*α* = 0.61 to *α* = 0.80) reported for the EAI in a cross-cultural investigation (Griffiths et al. 2015).

Passion was assessed with the revised Passion Scale (PS; Marsh et al. 2013) which measures harmonious passion (HP), obsessive passion (OP), on two 7-item subscales and passion criteria (PC) on a 5-item subscale. The latter is usually used as control for the validity of OP and HP. Its five items reflect the conceptualization of passion (Vallerand et al. 2003). For example, they mirror how much a person likes or loves the activity, appreciates it, dedicates time and energy to it, views part of her- or himself, and considers the activity passionate. While PC is not used as a dependent measure, it should be positively related to both OP (i.e., in the present study *r* = 0.57, *p* < 0.001) and HP (*r* = 0.67, *p* < 0.001) (Marsh et al. 2013; Vallerand et al. 2003). All its items are rated on a 7-point Likert scale, ranging from “not agree at all” to “very strongly agree”. A sample item for OP is “If I could, I would only do my activity”, for HP: “This activity is in harmony with the other activities in my life,” and for PC: “This activity is part of who I am.” Higher scores reflect higher passion on each subscale. Here, we used the Spanish version of the scale (Chamarro et al. 2015). The internal reliability (*α*) of the PS in the current work was 0.89, which is a higher value than that (0.81) reported by Chamarro et al. (2015). The internal reliabilities of the three subscales were as follows: OP = 0.83, HP = 0.82, and PC = 0.83. Given that it was repeatedly demonstrated that passion shares a large proportion of the variance with EAI scores (Kovacsik et al. 2019, 2020), its assessment was used for controlling the moderating effects OP and HP on the risk of exercise addiction.

We measured perfectionism with Frost Multidimensional Perfectionism Scale (FMPS; Frost and Marten 1990). This scale can be used as both unidimensional and multidimensional instrument. In the former case, 29 of 35 items are summed to provide an overall measure of perfectionism. The scale’s six subscales^1^, of which five evaluate dimensions of perfectionism, such as personal standards or doubt about one’s action, could be used for measuring the various components of perfectionism separately. However, in this work, we were interested in the moderating role of the unidimensional perfectionism on the risk of exercise addiction. A sample item is “I have extremely high goals.” All items are rated on a 5-point Likert scale. Six items, representing “organization” are not considered. After reversely rating some of the items, the individual ratings are summed up for 29 items to obtain a total score for perfectionism. In the present study, we employed the Spanish version of the FMPS (Gelabert et al. 2011). Its internal reliability in the current sample was 0.93.

### Procedure

After obtaining ethical clearance from the Autonomous University of Madrid, demographic questions and the three psychometric instruments were published on Google Forms survey platform. Volunteers were eligible to take part in the study if they were aged 18 years or over and exercised for at least 1 hour per week. The gathered data were checked and the answers of those who did not meet the criteria for participation were deleted from the dataset (*n* = 37). Prior to being able to see and answer the questionnaires, participants gave consent to participation by marking with a mouse- click an “agree” button at the end of the consent form. This form contained information about the study as well as criteria for participation. Data were collected between the 6th and 20th of April 2020 and were saved in an Excel file. Subsequently, this file was imported in the SPSS statistical software (version 26) which was used for data verification and analyses.

### Data Reduction and Analyses

The grouping of the risk of exercise addiction was performed after rating the EAI. Accordingly, a score of 24 or above reflects risk of exercise addiction, a score between 13 and 23 indicates moderate risk (symptomatic), and a score between 6 and 12 is indicative of low risk (asymptomatic) (Terry et al. 2004). Change in exercise volume was calculated by subtracting the current total weekly hours of exercise from that reported for the period before the COVID-19 pandemic. Descriptive statistics were used in determining the overall prevalence of the risk of exercise addiction in the current sample. Chi square (*χ*^*2*^) was used to examine the frequency of health, skill, and socially motivated exercise among the three exercise addiction risk groups. A bivariate and a partial correlation (controlling for passion [OP, HP] and perfectionism) were calculated to examine the relationship between exercise addiction risk scores and changes in exercise volume. Analysis of covariances (ANCOVA) was used in examining the change in exercise volume, as a result of the COVID-19 pandemic, in individuals at risk of exercise addiction having different primary motives for exercising as well as in those exercising in team and individual exercises. Further, an ANCOVA was also employed in examining the perceived impact of the COVID-19 pandemic on the training regimen of the three exercise addiction risk groups. In addition to gender and age, in all these analyses, we also controlled for passion as suggested by Szabo and Kovacsik et al. (2019), as well as perfectionism.

## Results

The overall decrease in habitual exercise was nearly 50% (49.24%) in the whole sample. Participants exercised an average of 9.22 (± *SD* = 5.65) hours/week before and 4.54 (± *SD* = 3.76) hours/week during the COVID-19 pandemic (*t*_[1078]_ = 29.84, *p* < 0.001, effect size Cohen’s *d* = 0.91). The proportion of those at risk of exercise addiction was 15.2%, those showing some symptoms amounted to 79.6%, and 5.2% were asymptomatic. The *χ*^*2*^ test yielded statistically no significant difference between the eight nations. However, a statistically significant distribution emerged in the observed frequency of the three primary exercise motives (health, skill, and social) in the three exercise addiction risk groups (*χ*^*2*^_[4]_ *=* 9.88, *p* = 0.043). As illustrated in Table 1, this result may be attributed to the different ratio of skill, but mainly social, reason in the asymptomatic group compared to the other two groups. Among all exercise addiction risk groups, health was the most frequently reported motive for exercise (81.7%).

**Table 1:** Frequency of exercise motives in three exercise addiction risk groups

EAI Classification	Health	Skill	Social
Asymptomatic (*n* = 56)	75%	8.9%	16.1%
Symptomatic (*n* = 859)	82.7%	11.5%	5.2%
At risk (*n* = 164)	79.3%	13.4%	7.3%
Total (*n* = 1079)	81.7%	11.7%	6.6%

**Fig. 1:**
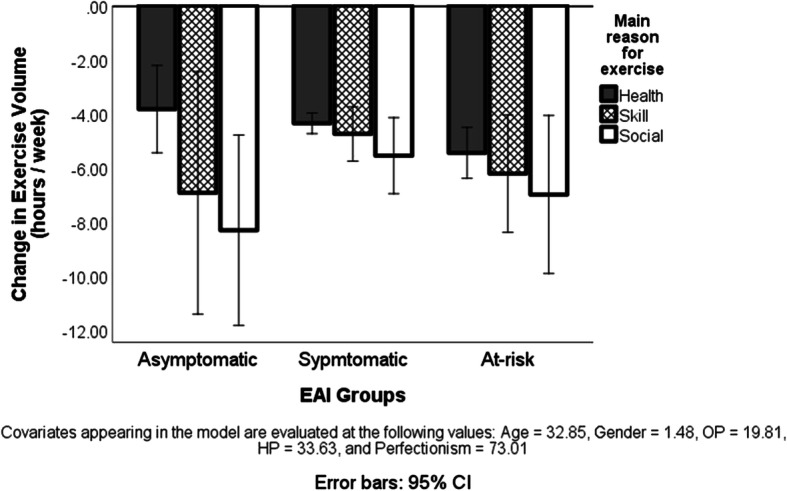
Change (decrease) in the weekly hours of exercise in the three exercise addiction risk groups, exercising primarily for health, skill, or social reason, as a result of the COVID-19 pandemic. Note: The value 0 on the *Y*-axis is the reference point (i.e., usual amount of exercise before the COVID-19 situation). CI, confidence interval; EAI, exercise addiction inventory based classification

To test the hypothesis that those at risk of exercise addiction, having a health-related primary motive for their exercise, exhibit the least change in exercise volume due to the COVID-19 pandemic, we calculated two ANCOVAs. The first only controlled for age and gender while the second also controlled for passion (both OP and HP). Further, because a statistically significant correlation emerged between EAI scores and perfectionism (*r* = 0.26, *p* < 0.001), we also controlled for perfectionism in the second model. The results of the first model yielded a main effect for exercise addiction risk groups (*F*_[21066]_ = 4.32, *p* = 0.013) and for primary exercise reason (*F*_[21066]_ = 5.74, *p* = 0.003), but no interaction between the two. At this early point, we disregarded the between-group differences, because we wanted to obtain the results of the second ANCOVA which also included passion and perfectionism in the model. This test was no longer statistically significant for the exercise addiction risk groups, (*p* > 0.05), but it was still significant for the primary reason for exercise (*F*_[21061]_ = 4.55, *p* = 0.011; see Fig. 1). The Bonferroni corrected post-hoc tests revealed that respondents exercising for health reasons decreased their exercise volume less than those exercising primarily for a social reason (*p* = 0.018), but neither of these groups differed from those who exercised primarily for a skill-related reason.

To test the relationship between the risk of exercise addiction and the change in weekly exercise volume, first we calculated a bivariate correlation, which was weak, but statistically significant (*r* = −0.127, *p* < 0.001). However, when a partial correlation controlling for passion and perfectionism was applied, as suggested in the literature (Szabo and Kovacsik 2019), the relationship was no longer significant (*r* = −0.024, *p* > 0.05). The zero-order correlations showed that exercise addiction was positively and statistically significantly related to all control variables (OP: *r* = 0.57, *r*^*2*^ = 0.32, *p* < 0.001; HP: *r* = 0.42, *r*^*2*^ = 0.18, *p* < 0.001; PC: *r* = 0.49, *r*^*2*^ = 0.24, *p* < 0.001; and perfectionism: *r* = 0.26, *r*^*2*^ = 0.07, *p* < 0.001). The result of the partial correlation justified the consideration of the second model in ANCOVAs which controlled for passion and perfectionism in the context of the risk of exercise addiction.

**Fig. 2:**
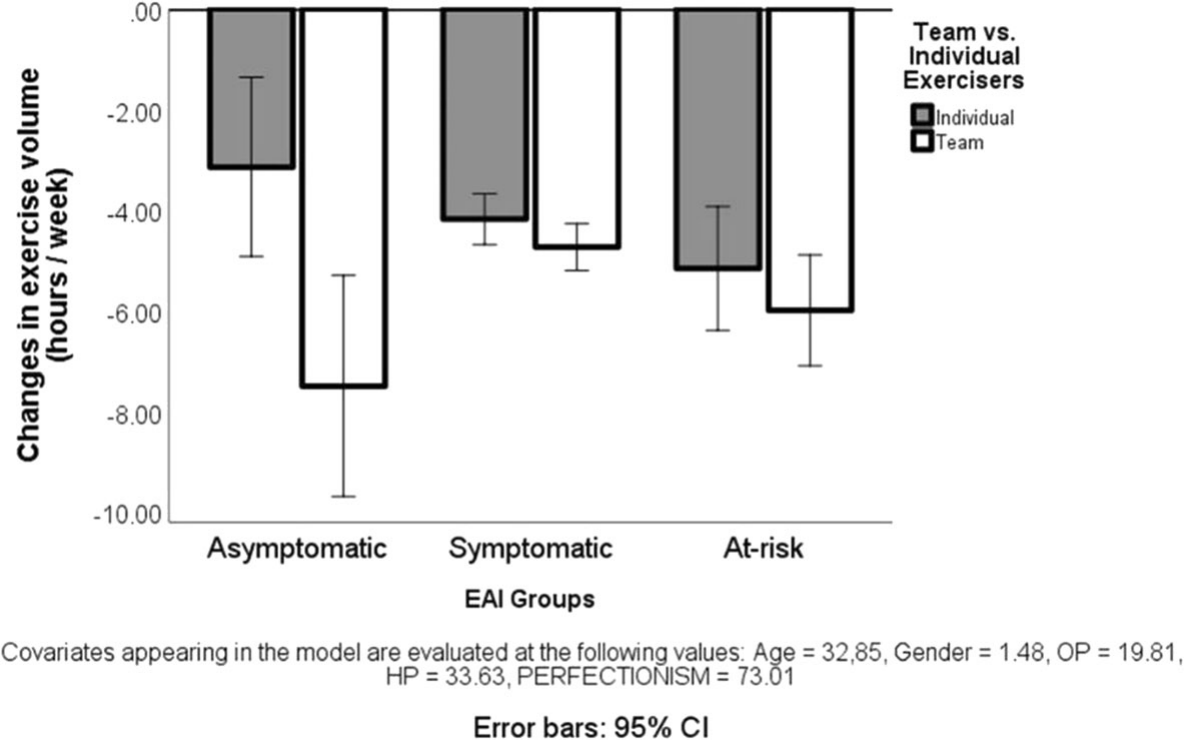
Change (decrease) in the weekly hours of exercise in the three exercise addiction risk groups because of the COVID-19 pandemic. Note: The value 0 on the *Y*-axis is the reference point (i.e., usual amount of exercise before the COVID-19 situation). CI, confidence interval; EAI, exercise addiction inventory based classification

The ANCOVA, testing the hypothesis that team/group exercisers at various risk levels of exercise addiction show greater decrease in exercise volume due to the COVID-19 pandemic than individual exercisers, yielded a statistically significant interaction when controlling for age and gender (*F*_[21069]_ = 3.80, *p* = 0.023), as well as when we added passion and perfectionism to the model (*F*_[21064]_ = 3.44, *p* = 0.033). This result was due to a significantly larger decrease in asymptomatic team exercisers than asymptomatic individual exercisers (see Fig. 2).

**Fig. 3:**
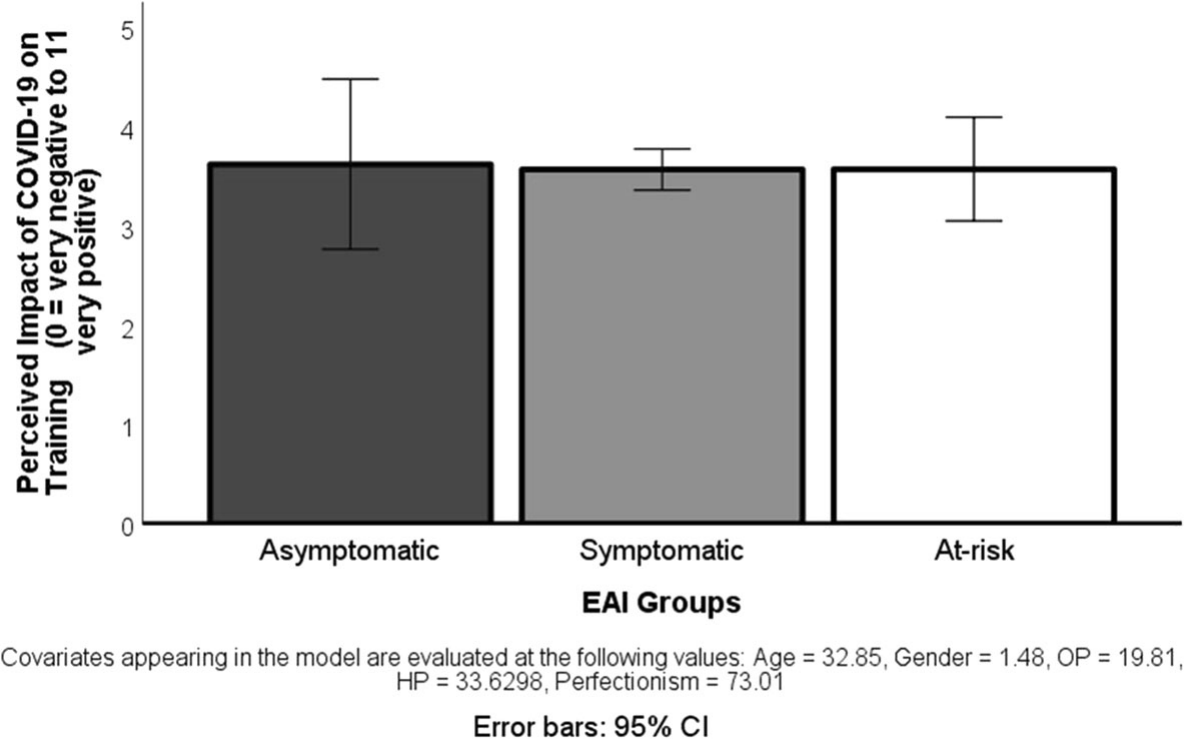
Perceived impact of the COVID-19 pandemic on the physical exercise training in three exercise addiction risk groups. Note: CI, confidence interval: EAI, exercise addiction inventory based classification

The ANCOVA, testing the hypothesis that those at risk of exercise addiction perceive a greater negative impact of the COVID-19 situation on their usual training regimen than the two other lower-risk groups, was statistically not significant (see Fig. 3).

## Discussion

The main contributions of the present international study are (1) the COVID-19 pandemic forces habitual exercisers to decrease their usual exercise volume to about half of the usual amount; (2) the majority of exercisers have a health-related primary reason for exercising; (3) if passion and perfectionism are not included in the model, there is a significant difference in the primary reason for exercise between the three exercise addiction risk groups, but when the model controls for these variables, the difference does not emerge anymore; (4) individuals exercising primarily for a health-related reason decrease less the volume of their exercise during the COVID-19 pandemic than those exercising for social reason; (5) a weak correlation between the decreased exercises volume during the COVID-19 pandemic and the risk of exercise addiction exists only if there is no control for passion; otherwise, the relationship is not significant; (6) only team exercisers in the asymptomatic exercise addiction risk group have decreased more their exercise volume than individual exercisers in the same risk group; (7) the three exercise addiction risk groups do not differ in their perceived impact of the COVID-19 pandemic on their habitual exercises.

A decrease to about half in the usual exercise volume could be ascribed to the COVID-19 pandemic as revealed by the results of this international study. At this time, there is no report available in the literature to which these findings could be compared. However, the here observed overall change in the habitual exercise volume may be considered “reasonable” based on limited movement, distance keeping, and closure of many exercise facilities in many nations during the COVID-19 pandemic. The current results pave the road for research on the physical and mental health consequences of this major cut-back in physical activity in an already sedentary world.

The decrease in exercise volume was initially statistically significant between the three exercise addiction risk groups, but when passion and perfectionism were added to the model, the three groups did not differ anymore. These findings support the strong connection between the concepts of risk of exercise addiction and passion (Kovacsik et al. 2019, 2020) as well as perfectionism (Bircher et al. 2017; Curran et al. 2014; Schipfer and Stoll 2015). Therefore, the results justify the recent call for considering passion, and based on the current study perfectionism too, in all research in which the risk of exercise addiction is a dependent measure (Szabo and Kovacsik 2019).

The decrease in exercise volume due to the COVID-19 situation was lower in health-oriented exercisers compared to those exercising primarily for a social reason. This finding only partially confirms our hypothesis, because people exercising mainly for a health reason did not differ from the skill-oriented exercisers. The least cut down in exercise volume in this group might be explained in light of the Health Belief Model (Rosenstock 1974). The gist of the model is that when a person feels threatened by a morbidity, which is determined by susceptibility and severity, and benefits need to be evaluated against the barriers, greater effort is invested in conquering the barriers when the perceived threat is greater. During the COVID-19 pandemic, exercise facilities and social exercising may be limited, but if there is a threat to the health, the person could find alternative ways to exercise. Therefore, in accord with our hypothesis, this group manifests the least decrease in exercise volume (i.e., overcomes the most the imposed barriers). Clearer distinction between health-motivated and other exercise reasons may become more evident over a longer period that can be investigated in longitudinal research.

In accord with our hypothesis, team exercisers decreased more their exercise than individual exercisers. However, when examining the decrease between the two groups in the context of the risk of exercise addiction, only asymptomatic team exercisers differed significantly from asymptomatic individual exercisers, with the former decreasing more than twice as much their exercise volumes due to COVID-19 pandemic than the latter. There was almost negligible difference (see Fig. 2) in the amount of decrease in exercise volumes in the other exercise addiction risk groups. It is possible that “usual” team exercisers who show some symptoms of risk of exercise addiction, or are at risk, compensate with individual exercises for the lost team exercise during the COVID-19 crisis. This explanation should be tested empirically in future studies. Regrettably, we did not ask the types of exercise performed before and during COVID-19.

Despite our hypothesis that those at risk of exercise addiction will perceive a greater negative consequence of the COVID-19 situation on their training than the others, the results did not support our conjecture. In fact, there was no difference in the perceived impact of the COVID-19 situation between the three exercise addiction groups (see Fig. 3). All groups reported a slightly negative perceived effect (the 5 is the median of the assessment scale). We interpret these findings as an outcome of a subjective appraisal of control conveyed in the responses, which is basically similar to all regular exercisers, regardless of their risk of exercise addiction.

Our hypothesis that there will be a negative relationship between the risk of exercise addiction and change in exercise volume during COVID-19 was supported only until we did not control for passion and perfectionism. After controlling for these variables, the relationship vanished, and all zero-order correlations were significant, supporting the large commonality between these factors (Szabo and Kovacsik 2019). Therefore, the current results, both those based on ANCOVA and correlation, lend support for the strong need to control for passion in exercise addiction research as suggested by Szabo and Kovacsik. However, our findings expand their results by showing that perfectionism is also instrumental in the risk of exercise addiction, and exerting control for this variable is also recommended. While the correlation between exercise addiction and perfectionism was statistically significant, the coefficient of determination (*r*^*2*^) was lower than the values obtained for passion, indicating that perfectionism shares less common variance with exercise addiction than passion. Nevertheless, the connection between exercise addiction and perfectionism seems to affect the results related to exercise addiction.

### Strengths and Limitations

The strength of the current work is that it is a pioneering study during COVID-19 in the context of exercise addiction reason for exercise and form of exercise, and it is based on large number of responses from eight Spanish-speaking nations. Still, another strength of the study is that it is the first, based on authors’ best knowledge, to examine the risk of exercise addiction in the context of primary reason for exercise. Last, but not least, the results of the study confirm in a large sample that the inclusion of passion and perfectionism in the evaluation models of the risk of exercise addiction is necessary, because without controlling for passion different results, encompassing different (but false) explanations, emerge. The limitations of the present study are manifested in the volunteerism, which is a general problem in psychological studies. The internal reliability of the EAI was 0.65, in the current study which is at the lower end of the acceptable range and, hence, this is another limitation of this work. Further, these results cannot be generalized to non-Spanish speaking samples and despite all eight nations were affected by COVID-19, not all of them were affected equally. Indeed, the level of lockdown differed between these nations (BBC.com, April 6, 2020) and due to the anonymous nature of the inquiry, we do not know from which exact local regions did the people respond, which is important because within the eight countries examined there were regions more or less affected by the pandemic. Therefore, our findings may be more pronounced in the heavily virus-affected areas whereas they may be less evident in regions less affected by COVID-19.

## Conclusions

Exercise volume decreases about 50% in regular exercisers 2 months into the COVID-19 crisis. The decrease and the primary exercise motive are unrelated to the risk of exercise addiction. Most exercisers report a health-related motive as the primary reason for their exercise and this motive is associated with lesser reduction of exercise volume than a social reason. During COVID-19 team exercisers decrease more their exercise volume than individual exercisers, but the difference is evident in the symptomatic exercise addiction risk group only. The findings confirm the call for controlling passion in exercise addiction research. They expand this call by showing that perfectionism could also moderate the results obtained on exercise addiction and, therefore, this variable should be also considered, along with passion, in future exercise addiction research.

## Footnotes

1 A version with four subscales also exists, but the psychometric properties of the six-subscale Spanish version are superior to that of four-subscale version.
